# POM@PMO plastic electrode for phosphate electrochemical detection: a further improvement of the detection limit

**DOI:** 10.1007/s00604-023-05679-1

**Published:** 2023-03-15

**Authors:** Sondes Ben-Aissa, Rossella De Marco, Sabina Susmel

**Affiliations:** 1 Bioanalytical Chemistry-Aquaculture and Wildlife Management, Department of Agri-food, Environment, and Animal Sciences (Di4A), University of Udine, Via Sondrio 2/A, Udine, Italy; 2 Chemistry Department, Molecular Sciences Research Hub, Imperial College London, London, UK; 3 Organic Chemistry-Chemistry Section, Department of Agri-food, Environment, and Animal Sciences (Di4A), University of Udine, Via del Cotonificio, 108 Udine, Italy

**Keywords:** Phosphate, Periodic mesoporous organosilica, Octamolybdate, Plastic electrode, Square-wave voltammetry, Wastewater, Seawater

## Abstract

The development of a highly sensitive electrochemical sensor (E-sensor) is described based on stand-alone plastic electrodes (PE) for phosphate detection, being an essential nutrient in the marine environment. The detection mechanism is based on the chemical affinity between polyoxomolybdate anions (POM) and orthophosphate to form an electroactive phosphomolybdate complex. The custom-made E-sensor was formulated with an organic octamolybdate derivative (TBA_4_Mo_8_O_26_) incorporated with periodic mesoporous organosilica (PMO) to obtain a significant improvement in the analytical performances of phosphate determination. This POM@PMO combination was found to be advantageous in the determination of low concentrations of phosphate in standard solutions ranging from 1 to 500 nM, using square wave voltammetry as the detection technique. This sensitivity enhancement can be attributed to the effect of hydrophobic PMO in loading more POM moieties, owing to its highly porous structure and charged shell. Consequently, the POM@PMO-PE sensor achieved a competitive sensitivity of 4.43 ± 0.14 *μ*A.nM^−1^.cm^−2^ and a limit of detection of 0.16 nM with good selectivity against silicates. Finally, seawater and treated wastewater samples have been tested to validate the sensor response in comparison to the official method of phosphate determination.

**Graphical abstract:**

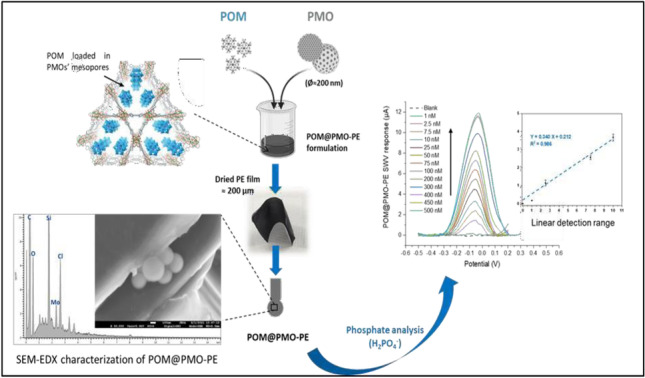

**Supplementary Information:**

The online version contains supplementary material available at 10.1007/s00604-023-05679-1.

## Introduction

Among the macronutrients, phosphorus P, mainly in the form of dissolved phosphate anions, has been adopted to rationalize the healthy state of the marine environment [1]. Oligotrophic seawater indicates an environment with limited phosphate concentration (10^−9^ M), frequently associated with an unbalanced Redfield ratio, which alters the fertility of the sea [2]. Therefore, several instrumental analytical techniques have been implemented for phosphate monitoring [3, 4]. The gold standard for phosphate detection in water is the spectrophotometric approach based on the formation of a phosphomolybdate complex in acidic conditions upon a reaction between phosphate and water-soluble molybdenum salts. This polyoxomolybdate complex (referred to as POM) is then reduced, usually by ascorbic acid, to transform into phosphomolybdenum blue, which is useful for the colorimetric detection of phosphate [5]. Despite its simplicity, this spectrophotometric method presents the drawback of requiring several steps of preparation, hindering thus its implementation for in-field use. Moreover, its results could be affected by the color or the turbidity of samples and by co-existing silicates highly present in seawater samples [6]. Likewise, chromatography and optical fluorescence have also been reported [7, 8], but such techniques often show low sensitivity, making them incompatible with the low concentrations of P in oligotrophic marine water. Therefore, cost-effective and highly sensitive tools are still needed for user-friendly on-site applications.

Alternatives based on potentiometric ion-selective sensors, as well as optical and electrochemical (bio) sensors, have been optimized [9–12]. For instance, Sivasankaran et al. [13] have recently developed an electrochemical sensor based on modifying gold electrodes by purposely synthesized dipicolylamine–zinc(II) complexes. It was demonstrated that E-sensors displayed high sensitivity toward phosphate with detection limits close to 1.0 × 10^–15^ M, allowing phosphate quantification in lake water samples. Phosphate biosensing based on enzymes [14, 15] has also been investigated. Still, the harsh conditions that often characterize environmental samples (i.e., temperature, composition, ionic strength, or pH) are sometimes detrimental to enzymatic activities. He and coworkers [16] recently designed an electrochemical biosensor based on pyruvate oxidase that catalyzes phosphate to produce hydrogen peroxide. The enzyme was immobilized on AuNRs@Cu_2_O-NDs and loaded on poly(diallyl-dimethylammonium chloride)-functionalized graphene at the surface of gold electrodes. The E-biosensor exhibited good performances in phosphate detection with a LoD of 0.4 nM.

Despite their advantages over classical approaches, such sensors suffer from a few limitations, including multistep fabrication processes and sensor instability due to the real matrix complexity. Besides, phosphate is neither optically nor electrochemically active to display an intrinsic signal. Furthermore, its sensitivity to pH makes the measurements even more challenging due to the eventual variations in the charge density and hydrophilicity of the anion.

In this context, our group recently developed a plastic electrode-based sensor, in which a molybdate derivative, tetrabutylammonium molybdate (TBA_4_Mo_8_O_26_ also known as POM), was intentionally synthesized to be soluble in the organic carbonaceous phase of the electrode composition [17]. It was thus possible to easily obtain stand-alone electrodes in which the Mo derivative was homogeneously distributed. This first generation of plastic electrodes enabled the electrochemical analysis of phosphate in seawater samples with a satisfactory detection limit of 5 nM [18].

For further improvements, it was interesting to examine the embedding of mesoporous materials in the PE formulation and investigate the properties of a nanostructured porous electrode material for phosphate sensing. Mesoporous materials are usually silica-based materials characterized by a high surface-to-volume ratio and a well-defined pore size so that the inner surface can be easily functionalized [18]. Several mesoporous silica families have been recently optimized, among which, periodic mesoporous organosilica (PMOs) are a class of organic–inorganic hybrids consisting of siloxane units bridged by organic units to form an ordered mesoporous framework [19, 20]. Generally, such ordered mesoporous organosilica are synthesized by hydrolysis and co-condensation of bifunctional organosiloxane precursors in the presence of a structuring agent in an acidic or alkaline environment. These PMO’s properties, including large internal surface, mechanical stability, and regular porosity, have led to their extensive application in host–guest incorporation, catalysis, selective separation, and sensing [21, 22]. An attractive feature of PMO materials is that the organic groups (R) are placed in the pore wall and are homogeneously distributed as an integral part of the inorganic-oxide framework, allowing higher loading of organic molecules [23]. Consequently, such an ordered scaffold improves both the charge and the mass transfer, thus enhancing the sensitivity of sensors and biosensors [19–23].

In the present study, periodic mesoporous organosilica coated with hydroxyl and amine terminal groups were synthesized and used to prepare PE sensors. An acidic solution of PMO and poly-oxo-molybdate (POM) was added to the bulk of carbon electrode formulation to obtain a new PE generation named as POM@PMO-PE.

Stand-alone carbon-based electrodes were obtained using a one-step process, overcoming the long stepwise modifications usually reported in the literature.

While amine-terminated PMOs presented a limited dispersion in the electrode formulation (data not shown here), POM@PMO-PE sensors based on hydroxyl-coated PMOs allowed phosphate quantification with an extended detection range from 1 to 500 nM. This strategy significantly improved the performance of the phosphate sensors by lowering the detection limit compared to our previous work [17]. The selectivity of E-sensors to phosphate has been assessed against silicate ions, known to be co-existing in water and reactive to POM with slower kinetics [6]. Finally, POM@PMO-PE sensors were applied in diluted real seawater and treated wastewater collected from Italian depuration plants. The results revealed reliable phosphate recoveries compared to the EPA-certified official method [23].

## Materials and methods

### Instrumentation and apparatuses

Scanning electron micrographs (SEMs) were collected with a Zeiss Auriga field emission scanning electron microscope instrument. IR spectra were recorded with a FTIR model Vector 22 (Bruker, USA) equipped with attenuated total reflectance (ATR) diamond crystal, with a resolution of 2cm^−1^ in the range between 4000 and 400cm^−1^.

Zeta potential measurements were performed with a Zetasizer Nano ZS (Malvern Panalytical, Malvern, UK), He-Ne laser 633nm, Max 4mW, using polystyrene cuvettes (optical path length 1 cm). Elemental analysis was performed with an EACE 1110 CHNOS analyzer (Thermo Fisher, USA).

All electrochemical measurements were carried out using Gamry Reference 3000 workstation (Gamry Instruments, Warminster, PA, USA). A homemade-designed plastic carbon electrode (PE) was set as a working electrode (WE) with a 3mm diameter of submerged area, along with a platinum counter electrode (CE) and an Ag/AgCl/KCl (3M) reference electrode (RE) using a conventional cell of 5mL. For in-solution studies, a glassy carbon electrode was used as the WE to assess the reactivity between POM and PMO in the presence of phosphate.

Cyclic voltammetry (CV) was recorded from −0.8 to 1V at 100 mVs^−1^ for characterization. Square wave voltammetry (SWV) was employed as the amperometric detection technique using the following optimized parameters: 25Hz of frequency, 1mV, and 25mV as potential step and potential pulse, respectively.

### Preparation of tetrabutylammonium octamolybdate TBA_4_Mo_8_O_26_ (POM)

The tetrabutylammonium octamolybdate was synthesized according to the protocol optimized by Klemperer [24], with slight modifications (*cf.* ESI for more details). For all structural and morphological characterizations, the reader is referred to our previous work [17, 25, 26] studying extensively this polyoxomolybdate (POM) material and its suitability to be embedded into plastic electrode formulation. Herein, only 1mg of TBA_4_Mo_8_O_26_ is used for electrodes preparation.

### Preparation of periodic mesoporous organosilica (PMO)

To prepare hydroxyl-coated POM nanoparticles, CTAB (484.5 mg, 1.33 mol) was dissolved in H_2_O/ethanol (90mL and 33mL), and 32wt% ammonia (0.075g, 1.2mL) solution was added. The reaction mixture was stirred at room temperature for 1 h before adding BTME (1.27g, 5 mmol) for organosilica nanoparticles’ preparation [27]. The above reaction mixture was continuously stirred for an extra time of 48h at room temperature. The CTAB mesoporous template was removed by stirring the sample in ethanol (50mL) with a 32Wt% aqueous solution of HCl (1.5g) at 50°C for 6 h.

**Scheme 1:**
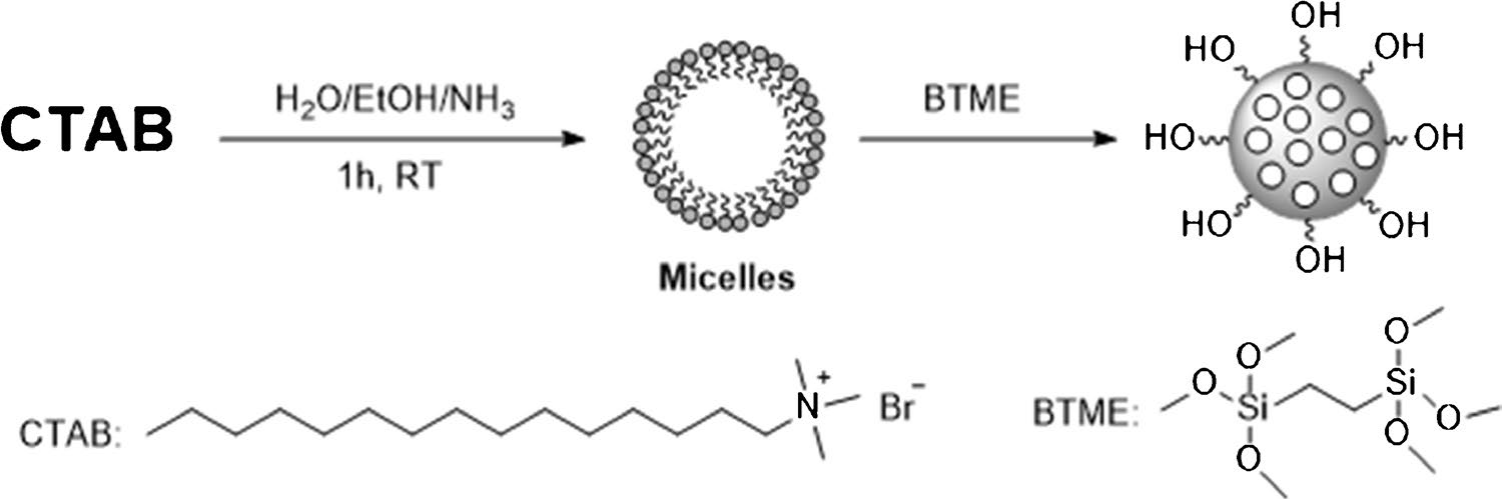
Experimental procedure for periodic mesoporous organosilica (PMO) preparation starting from CTAB [Created with BioRender.com].

The resulting solid was recovered by centrifugation (6000 rpm, 20min), washed with ethanol three times, and dried at 60°C under vacuum [20]. Here is reported the scheme of the reaction (Scheme 1).

### Preparation of modified plastic electrodes (POM@PMO-PE)

The plastic electrode decorated with octamolybdate anions embedded in mesoporous silica nanopores (POM@PMO-PE) was prepared as here reported. Briefly, 380mg of synthetic carbon graphite, 50mg of polyvinylchloride (PVC), and 50mL of bisethylexyl adipate (BEA) were successively dissolved in 9mL tetrahydrofuran (THF) solvent. Then, 1mg of POM dissolved in 200μL propylene carbonate (PC) was dropped after acidification by adding 20mM of H_2_SO_4_ to the mixture. Immediately, 1mg of PMO was added after a previous sonication in 1mL THF for 30 min. After thorough stirring in a humidity-controlled chamber at room temperature, the homogeneous slurry was distributed into a glass tray (8×6cm) and left to dry overnight under a medium-flux hood. The conductive film of 200μm thickness was cut with a custom paper punch and then fixed onto a PET flexible support (polyethylene terephthalate) to obtain miniaturized POM@PMO-PE of 0.25cm^2^ area (SI Fig. 8). Prepared electrodes were used directly or after storage in a low-humidity chamber containing silica gel.

### Orthophosphate analysis

The phosphate standard solutions were prepared by dissolving KH_2_PO_4_ in 0.1M KCl containing 1M H_2_SO_4_ as the buffer (pH < 2). A pre-incubation step was performed using 1 h of immersion in acidic buffer solution, to activate the electrode surface before phosphate analysis. The calibration was performed in blank solutions using anodic SWV measurements from −0.5 to 0.3V.

Three plastic electrodes for each concentration of phosphate standard solution were tested (*n*=3) to obtain final concentrations ranging from 1 to 500nM. The reproducibility was studied using measurements with three different electrodes in solutions containing 50nM of phosphate.

For selectivity measurements, two silicate solutions were prepared in the same conditions as phosphate standards to obtain final concentrations of 30 and 320nM.

### Real sample analysis

Samples were collected in June to July 2019 in the north of the Adriatic Sea, in Friuli Venezia Giulia Region (Italy), at the discharge points of the treatment plant of Lignano and San Giorgio. These pilot purifiers treat urban and industrial wastewater, respectively. The samples were collected at the end of the treatment process and before the discharging into the sea (WWT samples) and in marine water offshore (MW samples) close to the discharging point to avoid as much as possible dilution. All details about sampling procedures and analyses with official approaches and methods are reported in [28, 29]. The samples analyzed to validate the performance of POM@PMO-PE were collected in May 2019. Before analysis with PE sensors, WW samples were pre-treated in three steps: (i) sample filtration using 0.22-μm cellulose acetate filter, (ii) pH adjustment to 2 using 1M H_2_SO_4_, and (iii) dilution ×2500 folds in the working buffer (0.1M KCl, 1M H_2_SO_4_).

## Results and discussion

### Characterization of the POM@PMO-PE sensor

**Fig. 1:**
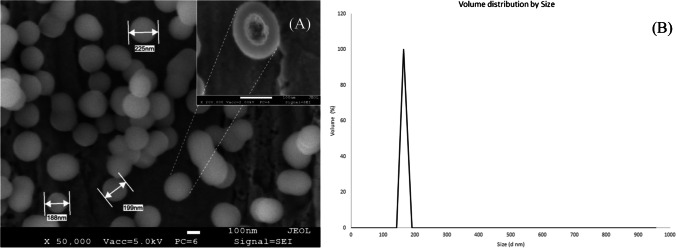
**A** SEM images of the synthesized organosilica particles PMO-OH at 50k of magnification (inset: 200k of magnification). **B** Size distribution by volume (H_2_O, 25°C) of PMO-OH nanoparticles measured by hydrodynamic light scattering (DLS)

Scanning electron microscopy (SEM) was first used to analyze the obtained hydroxyl-terminated PMO. Nicely formed organosilica nanospheres were evident in SEM images at a magnification of 50k (Fig. 1A). Increasing the magnification showed the core–shell structure of the nanoparticles (inset of Fig. 1A), confirming the obtention of the mesoporous structure (*cf.* FTIR spectra in ESI). An average diameter of 180nm was measured by dynamic light scattering (Fig. 1B). In addition, the zeta potential of about −17.6mV suggests a relatively good dispersibility of PMO-OH.

**Fig. 2:**
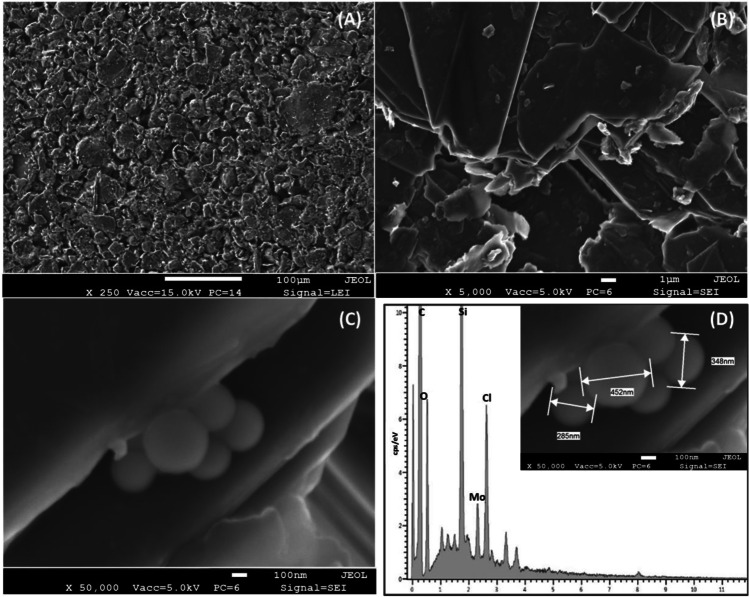
SEM images of the reactive side of POM@PMO-PE sensor before phosphate detection using different scales and orders of magnitudes **A** ×250, **B** ×5k, and **C** ×50k showing the embedment of PMOs nanoparticles in between carbon graphite micro-flakes and **D** corresponding EDX spectra

Once the modified PEs are prepared, the investigation of the distribution of POM@PMO in the hybrid carbonaceous film was also carried out by SEM coupled with energy-dispersive X-ray spectroscopy (SEM-EDX). The analyses here reported (Fig. 2) refer to POM@PMO-PE formulated using the hydroxyl-terminated nanospheres, highlighting the good embedment of PMO nanoparticles between carbon graphite micro-flakes (Fig. 2C). Moreover, the organosilica particles retain their intact structure in the PE formulation after solvent evaporation without a significant shape deformation (Fig. 2D) even if PMOs tend to slightly aggregate while being embedded between carbon graphite micro-flakes (diameter ≈ 350nm, about twice the initial diameter obtained with DLS and SEM results).

Considering the high acidity of the electrode environment to maintain molybdate reactivity (1M sulfuric acid), the shells of PMO are protonated (POM-OH_2_^+^). Therefore, we assume that the octamolybdate anions are preferentially attracted by the charged PMOs, which decreases the repulsion between PMO nanospheres and contributes to their mild aggregation via electrostatic interactions. It is also argued that POM anions could be loaded inside the organosilica mesopores by organic affinity, as already described by Zhou et al. [30], to prepare POM-based heterogeneous catalysts. Hence, the ion pairing is mainly supported by molybdate, which interacts preferentially with nanoparticles instead of its TBA counter-cation.

This observation seems confirmed by the EDX analysis (Fig. 2D), where silicon, carbon, and molybdenum elements are identified to be in the same electrode region. The oxygen is attributable to both POM and PMO structures, while the chloride source could be from the PVC polymer as the main film scaffold.

### Phosphate detection

The mechanism of phosphate determination in solution using polyoxometalates in acidic conditions is based on two consecutive reactions, (i) the formation of phosphomolybdate (PMo_12_) by condensation and (ii) the electrochemical reduction of Mo(VI) in PMo_12_ in the presence of protons [6, 31]:

1 $$3\;{\mathrm{Mo}}_8{\mathrm O}_{26}{}^{4-}+2\;{\mathrm H}_2{\mathrm{PO}}_4{}^-+8\;\mathrm H^+\Leftrightarrow2\;{\mathrm{PMo}}_{12}{\mathrm O}_{40}{}^{3-}+6\;{\mathrm H}_2\mathrm O$$

2 $${\mathrm{PMo}}_{12}{\mathrm O}_{40}{}^{3-}+2\mathrm n\;\mathrm H^++2\mathrm n\;\mathrm e^-\Leftrightarrow{\mathrm H}_2\mathrm n\;{\mathrm{PMo}}_{12}{\mathrm O}_{40}{}^{3-}{}$$

The reactivity of POM@PMO nanocomposite toward phosphate was first tested in solution using electroanalytical methods (cf. Fig. S5). Briefly, cyclic voltammetry (CV) was performed in propylene carbonyl solvent using a conventional glassy carbon electrode (GCE) as the working electrode (WE) to reveal a new peak for the phosphomolybdate formation at about −0.08V vs. Ag/AgCl reference electrode (cf. more details in ESI).

**Fig. 3:**
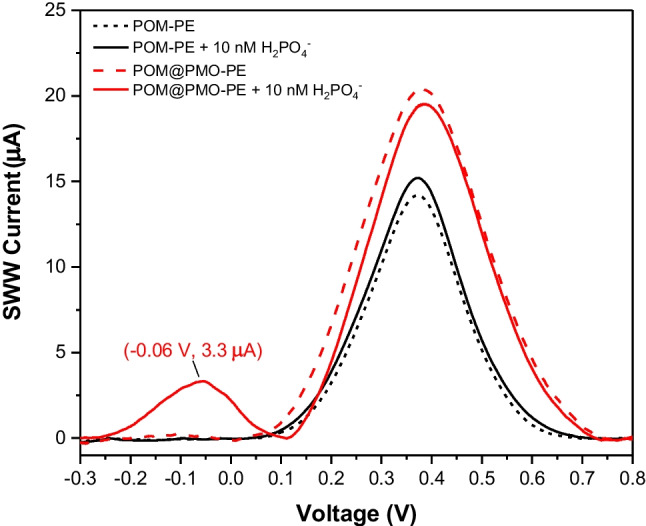
Comparison between the anodic SWV response POM-PE and POM@PMO-PE in 1M H_2_SO_4_ prepared in KCl 0.1M (black-dashed curves) and after adding 10nM of phosphate as NaH_2_PO_4_ (solid curves)

For the POM@PMO-PE sensors, this anodic process was studied by square wave voltammetry (SWV), given its better sensitivity than CV by enhancing the signal-to-noise ratio. Figure 3 compares the SWV signals of POM-PE and POM@PMO-PE, showing an enhanced sensitivity of octamolybdate in the second generation of sensors. Both sensors display a comparable oxidation peak at +0.4V with a 1.4-fold higher intensity when the POM@PMO is used. Interestingly, when 10nM of phosphate (H_2_PO_4_^−^) was added, the POM@PMO-PE sensor showed a new peak of about 3.3*μ*A at −0.06V with a slight decrease of the blank peak at −0.4V, which suggests the successful formation of PMo_12_. This OFF/ON signal around −0.06V was observed with only 5 min of reaction time under thorough stirring with good stability over analysis time. We then assume that octamolybdate electroactivity differs before and after complexation with phosphate in the case of POM@PMO-PE sensors. This behavior highlights the effect of PMO moieties in tuning the reactivity between octamolybdate and phosphate. The nanostructured organosilica particles seem to allow a better distribution of POMs as phosphate-capturing probes, throughout the electrode surface. In addition, the high rugosity of PE electrodes (Fig. 2B) in synergy with PMO properties gives the sensor a high surface-to-volume ratio and, therefore, a high conductivity, enhancing thus the electrochemical signaling.

**Fig. 4:**
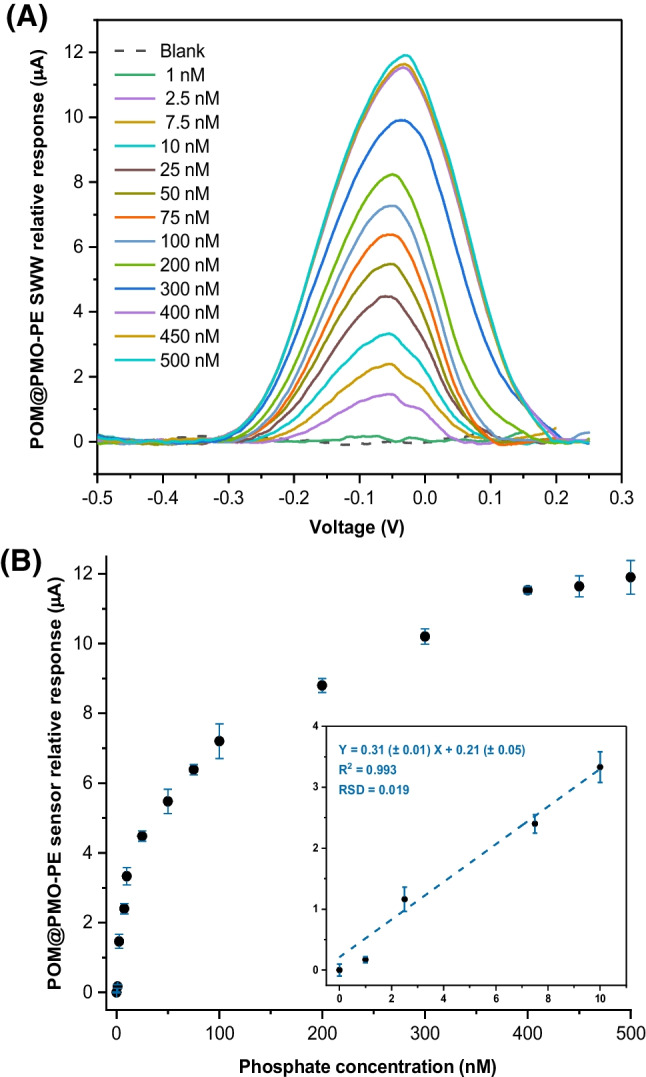
**A** Anodic SWV response of activated POM@PMO-PE sensors upon successive additions of aqueous H_2_PO_4_^−^ standards prepared in 1M H_2_SO_4_/0.1M KCl (blank) to obtain increasing concentrations 1–500nM. **B** Calibration curves were obtained based on the relative variation of the maximum peak intensity corrected by subtracting any blank noise (Ip–I_0_) at the corresponding potentials (−0.06 to −0.05V) as a function of cumulative phosphate concentrations (n=3)

Consequently, the POM@PMO-PE sensor has been used to analyze phosphate by adding an increasing amount of H_2_PO_4_^−^ to the aqueous buffer (0.1M KCl, 1M H_2_SO_4_) to reach final concentrations ranging between 1 and 500nM, i.e., 0.1 and 48ppb (Fig. 4A). This low concentration range was tested to target oligotrophic water samples in the framework of the Adswim project (ID 10046144). The results shown in Fig. 4 demonstrate the proportional increase of the anodic SWV peak with phosphate concentration accompanied by a slight positive shift in the oxidation potential, indicating the formation of the PMo_12_ complex. We note here that octamolybdate could not leach from the PE to the solution during measurements, since it is not water-soluble and is chemically embedded in the PE formulation.

Further experiments have been performed using POM@PMO-PE based on 2:1 POM:PMO ratio. However, the mediocre dispersibility of organosilica nanoparticles in the electrode formulation when exceeded 1mg resulted in an uneven electrode surface, with a bad distribution of the nanocomposite, which altered the sensors’ batch-to-batch reproducibility. The reader is referred to the ESI for more details.

The relative SWV response was found to be linear in the range of 1–10nM following the equation I (*μ*A) = 0.31 (± 0.01) [H_2_PO_4_^−^] + 0.21 (± 0.05). Therefore, the sensor metrics have been extracted from this linear calibration curve to obtain a sensitivity of 4.43 ± 0.14*μ*A.nM^−1^.cm^−2^ (slope/electroactive area), a limit of detection of 0.16nM (3*σ*_Blank_), and a limit of quantification of 0.53nM (10*σ*_Blank_). Hence, the sensitivity of POM@PMO-PE sensors has been significantly enhanced compared to the first generation of POM-PE sensors (LoD = 5nM) [17]. The new sensors have also shown better reproducibility based on an average relative standard deviation of 1.9%.

This improvement in the sensing performances confirms the abovementioned observations of POM@PMO interactions with phosphate in solution. The mesoporous silica nanostructure affords suitable nanocarriers to boost the formation of PMo_12_, owing to its large interfacial area between the aqueous phosphate and organic polyanions immersed in the hydrophobic layers, which enables the efficient diffusion of reactants to the active sites. Overall, the OH-terminated PMO afforded paths for the efficient recruitment of phosphate, water molecules, and protons to the octamolybdate anions sites. Accordingly, organosilica was found to increase the electrode wettability (Fig. S7) and then decrease the hydrophobic effect of TBA cations previously observed [17]. This exceptional behavior of organosilica was previously observed for catalysis applications [32–34]. In addition, the TBA counter-cation might be attracted to the aliphatic chains that form the bridging framework of the PMO so that the octamolybdate is more exposed to react with the boundary layer of orthophosphate molecules at the electrode interface, as comparably described in the functioning of ion-selective electrodes [35].

We should mention here that POM@PMO-PE sensors are disposable and are not considered regenerable at this stage because of the irreversible formation of the phosphomolybdate complex at the electrode. Nevertheless, the low cost of such material makes it suitable for fabricating single-use electrodes with good scale-up potential. It is also worth mentioning that the POM@PMO-PE sensors could be stored for up to 6 weeks in a low-humidity chamber without a substantial decrease in performance.

### Sensor selectivity

Based on our recently published data [28, 29], the co-existing interferents in oligotrophic water that could possibly be complexed with molybdate are mainly metal ions (arsenic) and silicate ions. Yet only silicates are present in a significant percentage compared to phosphate for the samples collected for this study (traces of As). SiO_4_^4−^ competes with H_2_PO_4_^−^ to react with molybdate ions, given their similar tetrahedron-shaped structure [5, 6]. Nonetheless, the kinetics of POM reaction with silicate is known to be substantially slower than phosphate [36]. Advantageously, the suggested detection protocol relies on a 5-min analysis time to overcome such interference.

Hence, the selectivity of POM@PMO-PE sensors was tested against silicates at two different concentrations, selected to be in the lower and higher limits of the dynamic detection range, namely, 33nM and 320nM.

**Fig. 5:**
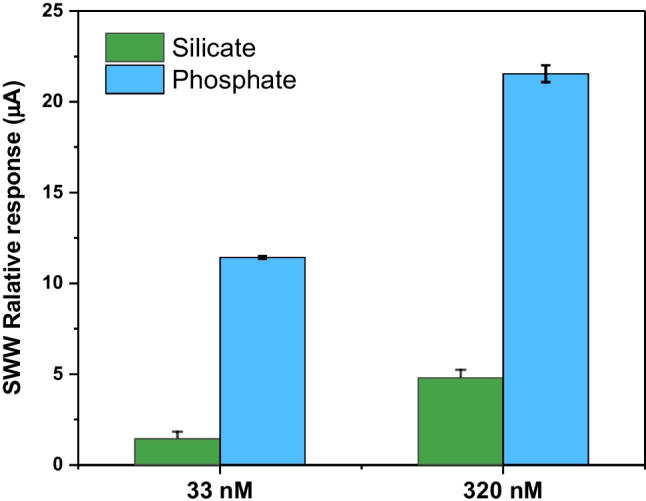
Anodic SWV response of activated POM@PMO-PE sensors toward two different concentrations of phosphate and silicate as a potential interferent. The error bars are the RSD of three independent measurements (*n*=3)

As shown in Fig.5, the electrochemical response of phosphomolybdate was at least fivefolds higher than the silicomolybdate at the same concentration. Moreover, the incubation of a tenfold excess silicate compared to phosphate (320nM SiO_4_^4−^ vs. 33nM H_2_PO_4_^−^) showed a favored reactivity to phosphate with a doubled SWV response. Therefore, POM@PMO-PE demonstrated good discrimination of phosphate against co-existing silicate.

### Real sample analysis

To assess the sensor’s reliability, oligotrophic water samples of known phosphate concentrations [29] have been analyzed following the same procedure described for standard solutions. No spiking has been used for this analysis, given the low LoD achieved with POM@PMO-PE sensors. The samples were subject to mild 3-step pretreatment (filtration, acidification, and dilution) as described in the “Experimental” section. Therefore, the SWV response was registered and applied to the linear calibration equation obtained with standard solutions to find the corresponding phosphate concentration. The latter was then multiplied by the dilution factor ×2500 to obtain the real amount of phosphate in the parent concentrated sample. Consequently, the recovery percentage was calculated to compare the E-sensors results to the data obtained by the conventional analytical method (EPA-certified method 45268), as shown in Table 1.

**Table 1:** Analysis results of treated wastewater and seawater samples: comparison between phosphate concentrations obtained with the POM@PMO-PE sensor and the official analytical method of nutrients analyzer based on recovery percentages

Sampling point ^[a]^	Nutrient analyzer (μM) ^[b]^ (A)		POM@PMO-PE sensor (μM) ^[c]^ (B)		Recovery (%) (B/A) x 100	
	WWT^[d]^	SW^[d]^	WWT	SW	WWT	SW
Lignano	5.22 ± 0.56	0.43±0.04	4.50± 0.38	0.42 ± 0.38	86.2	97.7
San Giorgio	1.66 ± 1.31	1.25±0.01	1.90± 0.18	1.20 ± 0.18	114.5	96.0

^[a]^Sampling period 05/2021 and Sampling area Friuli Venezia Giulia Region (IT). ^[b]^In Fanelli 2021. ^[c]^Dilution factor 2500, ^[d]^*WWT* treated wastewater, *SW* seawater

A good correlation between the sensor response and the nutrient analyzer method was obtained based on phosphate recoveries between 86 and 114.5%. Such satisfactory percentages highlight the applicability of the new E-sensor in both seawater and treated wastewater, making it a reliable and fast device to complement conventional analysis.

### Sensor benchmarking

Finally, a survey of the relevant literature, summarized in Table 2, confirmed the competitiveness of the POM@PMO-PE sensor compared to previously reported voltammetric E-sensors (excluding biosensors).

**Table 2:** Comparison of the analytical parameters of previously described electrochemical phosphate sensors

Sensor composition	Method	Linear range	LoD (nM)	LoQ (nM)	Sensitivity (*μ*A.nM^−1^)	Reagent addition	Analysis time (min)	Ref.
POM@PMO-PE	SWV	1–10nM	0.16	0.53	0.31 ± 0.01	H_2_SO_4_	5	**This study**
POM-PE	SWV	6–150nM	5	6	0.104	H_2_SO_4_	< 5	[17]
Molybdate-modified carbon paste electrode	SWV	0.01−5μM	3	10	0.014	NaOH	1.5	[37]
Molybdenum blue-modified PGE	DPV	0.025–1μM	21.9	73	0.003	H_2_SO_4_	60	[38]
Paper SPE/carbon black	CV	10–300μM	4 × 10^3^	10 × 10^3^	0.001	Mo_7_O_24_^6−^, H_2_SO_4_, and KCl	-	[39]
Rotating gold disk electrode	DPV	0.65–3μM	0.19 × 10^3^	0.65 × 10^3^	0.008	Tris buffer	18	[40]

The POM@PMO-PE sensors present a highly sensitive alternative to other techniques for the analysis of phosphate in the low nanomolar range. It showed a good compromise between the analytical performances and response time with promising potential for on-site application.

## Conclusion

This work described the successful embedding of octamolybdate anions with periodic mesoporous organosilica nanosphere in a new generation of PE-based sensors for phosphate detection. The use of POM@PMO nanocomposite showed a significant improvement in the analytical performances compared to the first generation of E-sensors previously reported without PMO. Therefore, we assume that hydroxyl-terminated PMO offers the advantage of preconcentrating the POM derivatives by organic affinity and electrostatic attraction. Besides their competitive sensitivity, the POM@PMO-PE sensors demonstrated adequate applicability in diluted real samples with minimal cross-reactivity to silicates within the optimal analysis time (5min). The developed proof-of-concept was promising to implement rapid phosphate detection tools for on-site analysis. However, the restricted linear detection range makes it more suitable for oligotrophic waters, i.e., poor in phosphate. Further investigations are still needed to state the mechanistic interactions between the organic POM, PMO, and carbon graphite within the PE for new applications. Finally, these results also highlight the flexibility in tuning PE-based formulations for other diagnostic applications based on specific chemical interactions.

## Supplementary information

ESM 1
