# Supplementary material for: POM@PMO plastic electrode for phosphate electrochemical detection: a further improvement of the detection limit
Source: Mikrochim Acta. 2023 Mar 15;190(4):135. doi: 10.1007/s00604-023-05679-1 (PMC10017643; doi:10.1007/s00604-023-05679-1)
Supplement: Supplementary file 1 — ESM 1 [file 604_2023_5679_MOESM1_ESM.docx]

**Electronic Supplementary Information**

POM@PMO Plastic Electrode for Phosphate Electrochemical Detection: A Further Improvement of the Detection Limit

Sondes Ben-Aissa^1,†^, Rossella De Marco^2^, Sabina Susmel^1^,*****

1. Bioanalytical Chemistry-Aquaculture and Wildlife Management, University of Udine, Department of agrifood, environment, and animal sciences (Di4A), Via Sondrio 2/A, Udine, IT. <http://orcid.org/0000-0002-6916-7373>.

2. Organic Chemistry-Chemistry Section, University of Udine, Department of agrifood, environment, and animal sciences (Di4A), Via del Cotonificio 108, Udine, IT. <http://orcid.org/0000-0001-7545-5921>.

† Chemistry Department, Molecular Sciences Research Hub, Imperial College London, UK. <https://orcid.org/0000-0002-4438-1760>.

* Sabina.Susmel@uniud.it

1. **Preparation of tetrabutylammonium octamolybdate TBA_4_Mo_8_O_26_**

Briefly, once the reaction between Na_2_MoO_4_ 2H_2_O and TBA-Br is achieved in acidic conditions (Figure S1), the octamolybdate powder requires thorough washing, especially with acetone, until the filtrate turns from yellow to clear to totally discard the excess of by-products, including other forms of polyoxomolybdates. Then, after cold crystallization in acetonitrile, transparent crystals were obtained and dried in a desiccator for 24h until a white TBA_4_Mo_8_O_26_ powder was formed as a final product. Using FTIR spectroscopy, spectra comparison to a previously synthesized batch revealed the high reproducibility and stability of TBA_4_Mo_8_O_26_ over time if properly stored in dry conditions at room temperature.


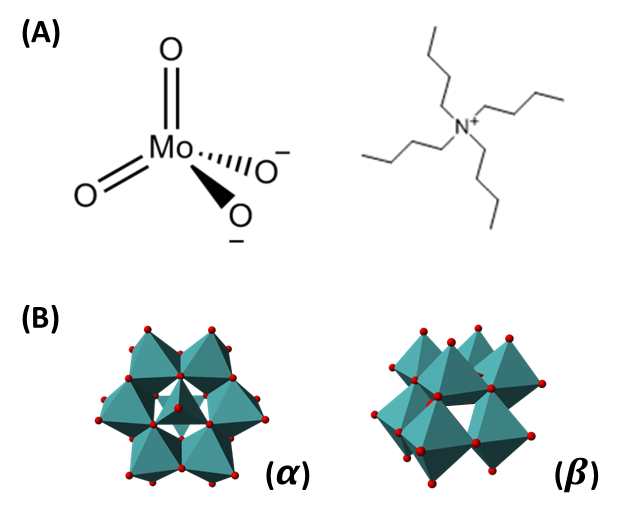


**Figure S1.** Tetrabutylammonium octamolybdate **(A)** chemical structure [N (C_4_H_9_)_4_]_4_Mo_8_O_26_ and **(B)** its Kegging structures (𝞪 and 𝜷 forms are coexisting).

1. **Characterization of obtained PMOs**

The nanoparticles were characterized with DLS, zeta potential, and IR.

The functionalized amino particles we synthesized showed a zeta potential of ca +15 mV, which suggests a marked hydrophilic character and an average diameter about four times with a wide dispersion of the size itself (ca +/- 180 nm) (Figure S2).

**Table S1.** There zeta potential values are reported of the PMO

| **NPs** | **ZP, mV** |
| --- | --- |
| PMO-NH_2_ | +15 |
| PMO-OH | -17.5 |


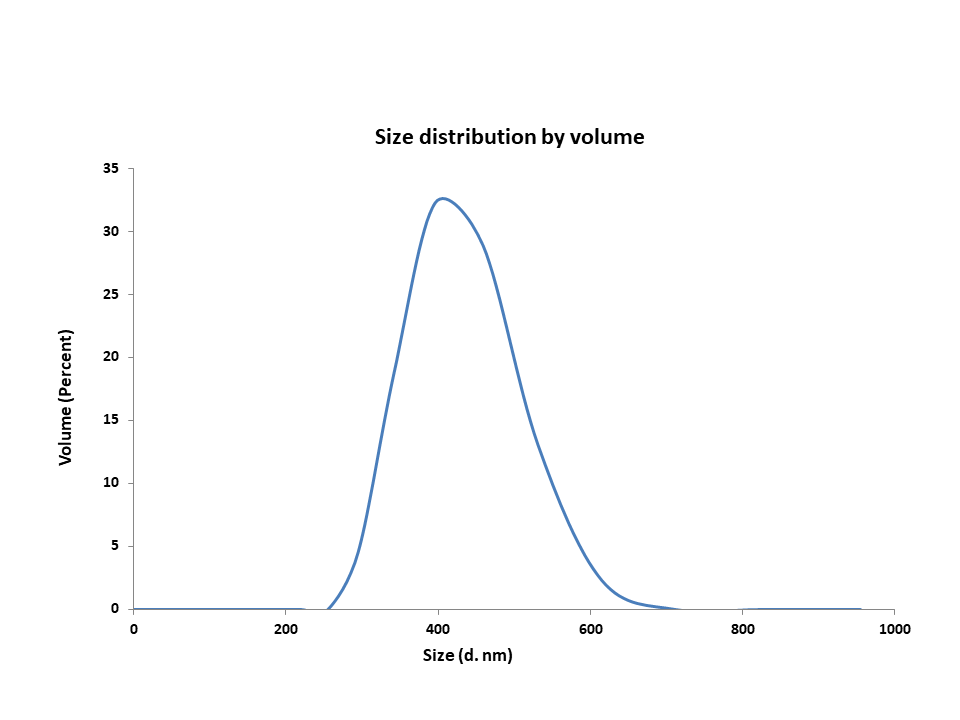


**Figure S2.** DLS hydrodynamic size distribution by volume (water, 25 °C) of PMO-NH_2_


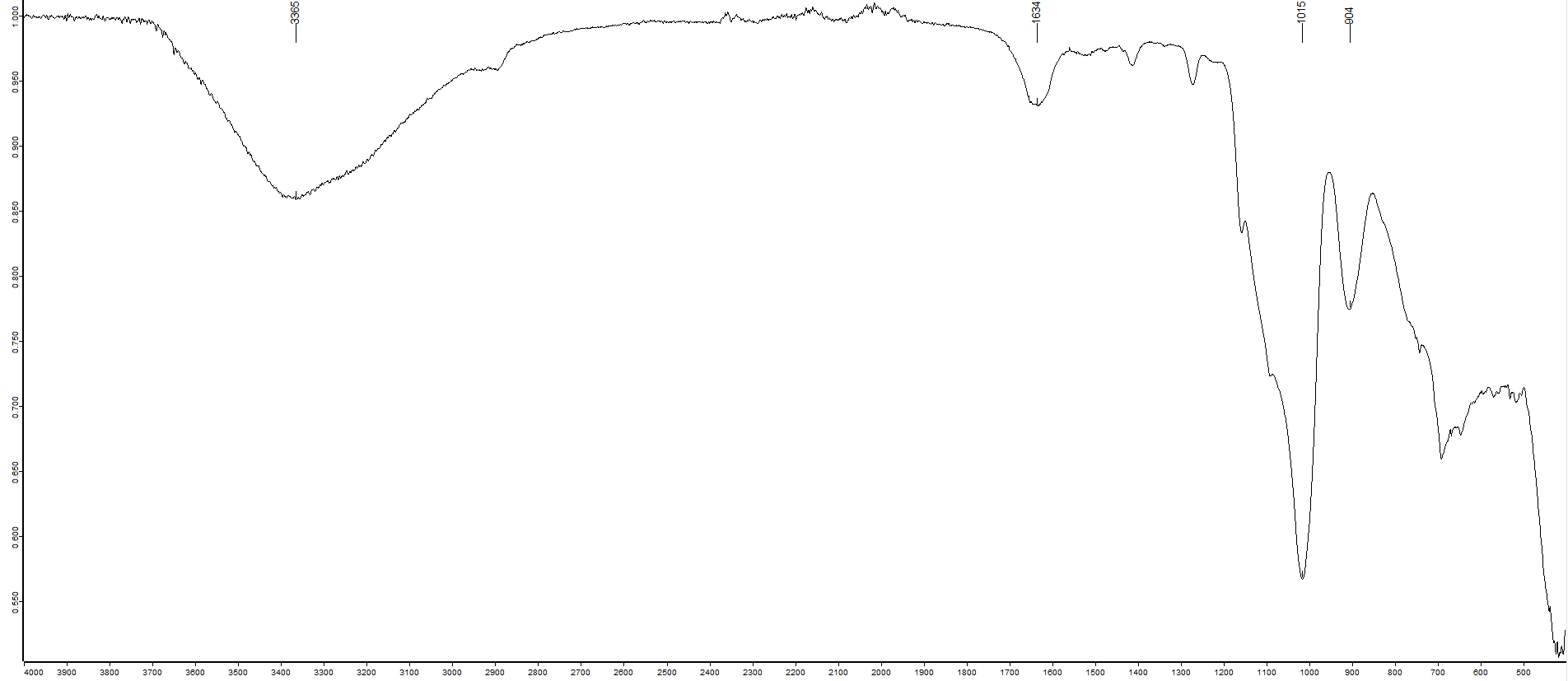


**A**


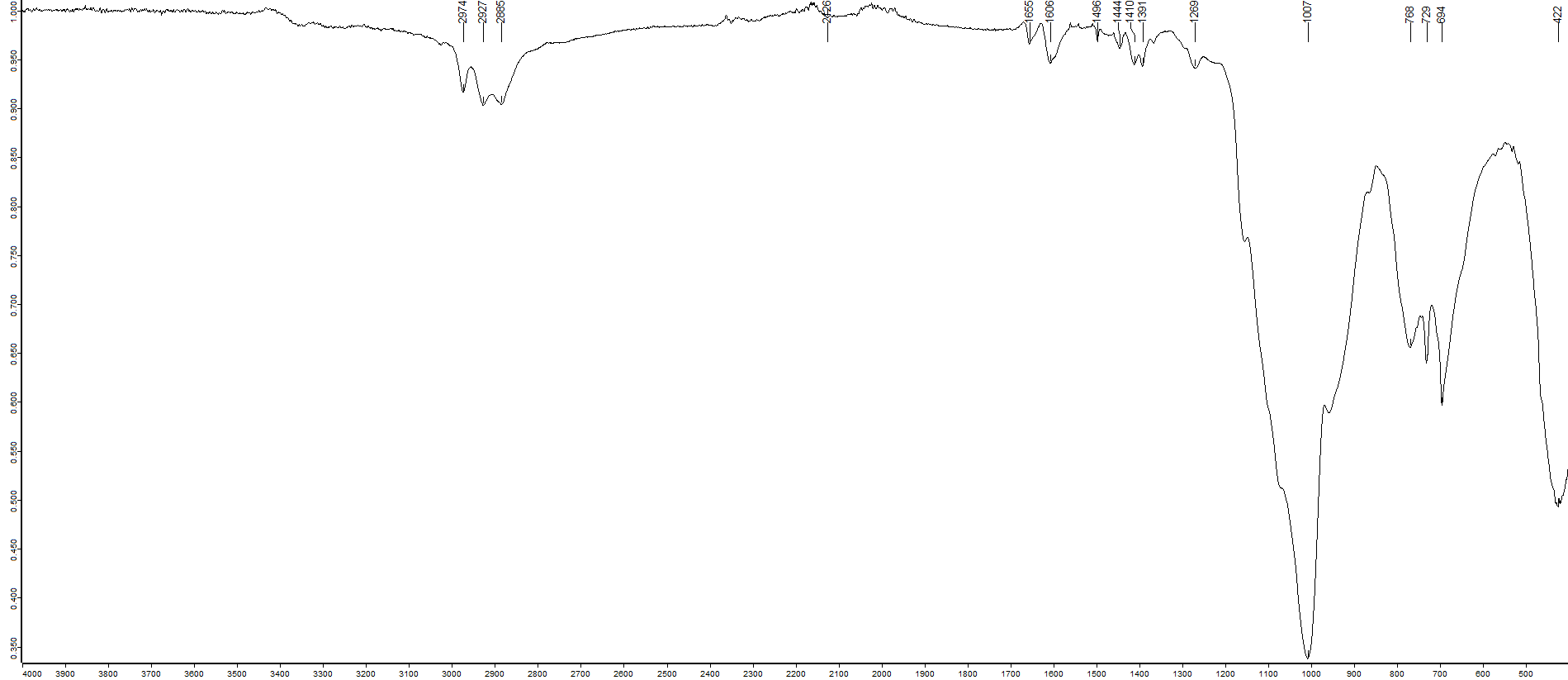


**B**

**Figure S3.** FT-IR spectrum of PMO-OH (A) and PMO-NH_2_ (B)

Figure S3 shows the AT-IR spectrum of PMO-OH and PMO-NH_2_. In the figure S3.A, the strong band at 3364 cm^-1^ corresponds to –OH stretching vibration of the silanol. The presence of a strong band at ca. 1014 cm^-1^, represents the asymmetric vibration of the siloxane bond, Si–O–Si. The band at 904 cm^-1^ corresponds to the stretching vibration of Si–O–Si bond. In figure S3.B, the presence of a band at ca. 2973 cm^-1^ corresponds to –NH stretching vibration, and the strong band at 1006 cm^-1^ showed the presence of asymmetric vibration of the siloxane bond, Si–O–Si.

1. **PMO and POM reactivity in solution**

The absorbance of POM at 360 nm was measured in THF in the presence of H_2_SO_4_, and this signal increased after the addition of PMO to the solution (Figure S4). This evidence corroborates the hypothesis that PMO and POM interact. We thus believe that PMOs tend to pre-concentrate POM moieties by organic loading inside their mesopores.

**Figure S4.** UV-Vis spectroscopic characterization of the PMO and POM reactivity in THF solvent containing 1M H_2_SO_4_ (Blank) per analogy to the PE formulation.

The cyclic voltammograms were recorded using pre-polished GCE prepared in propylene carbonyl (PC) solvent added with 1M H_2_SO_4_. As previously discussed, ^2, 8^ the high protons concentration is mandatory to ensure the octamolybdate anion reactivity towards orthophosphate. Moreover, the stepwise additions of POM, PMO-OH, and orthophosphate were investigated to control the octamolybdate behavior with different additives in organic phase. Figure S5 shows oxidation peaks at -0.2V, 0.38V, and 0.75V when only TBA_4_Mo_8_O_26_ is added. This is not surprising as the multivalent redox activity of Mo (VI) in polyoxomolybdates has been described in the literature owing to their fast structural rearrangements and sensitivity to the proton’s ratio ^8-11^.


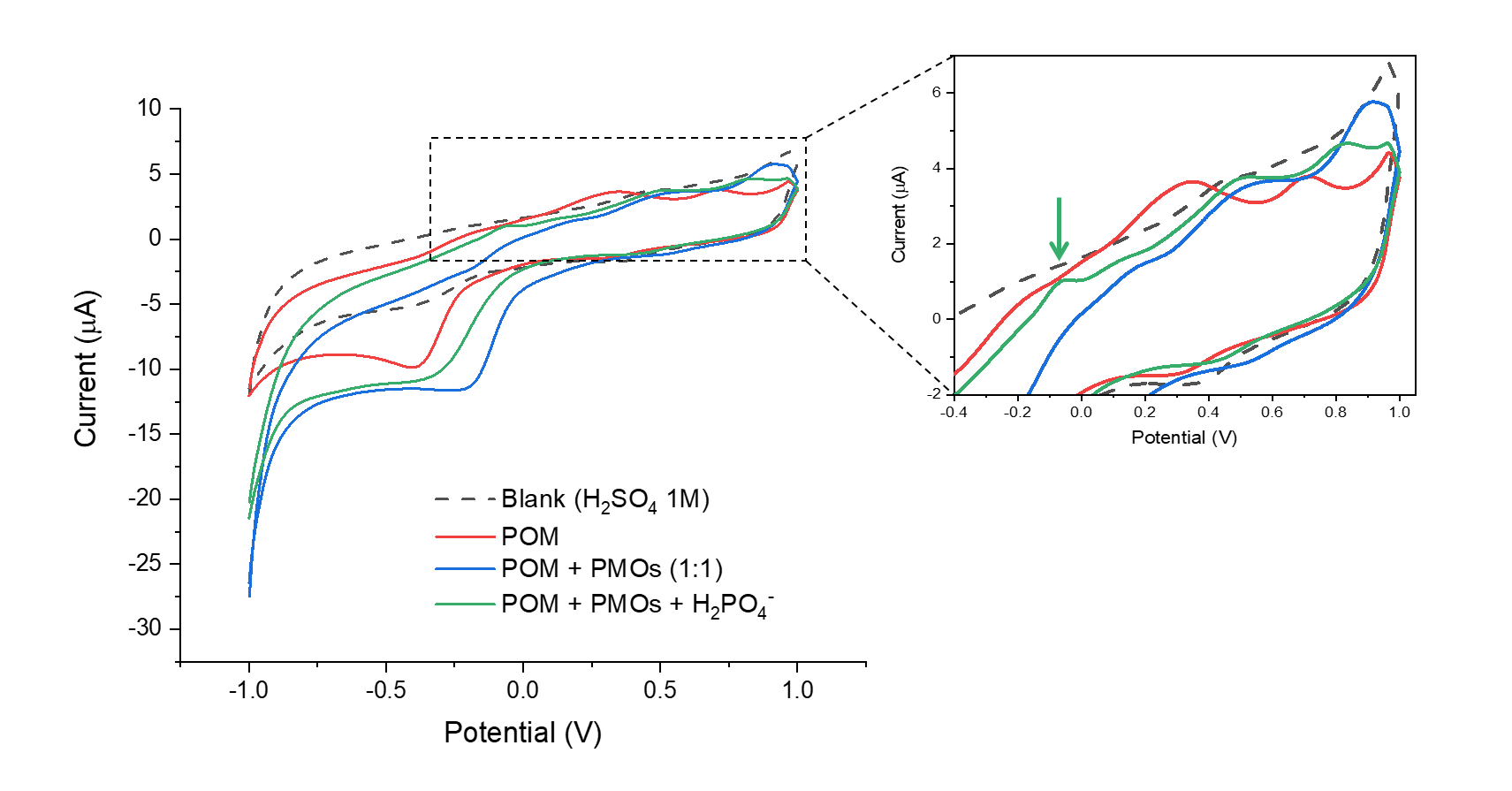


**Figure S5.** In-solution CV characterization of the TBA_4_Mo_8_O_26_ (POM) redox activity at 0.5 mM dissolved in PC containing 1M H_2_SO_4_ using GCE at 50 mV.s^-1^ scan rate (blank in dashed line). Voltammograms were recorded before and after the stepwise additions of POM (red), PMOs (blue), and TBA-H_2_PO_4_ (green) in the same solution at an equivalent 1:1 ratio. (Inset) Zoom on the anodic scans revealing a new peak for the phosphomolybdate formation in solution. The reader is referred to the electronic version for curves’ colors.

However, an anodic potential shift and current increase are both observed after the addition of organosilica particles in solution at a 1:1 ratio, suggesting the POM-PMO reactivity in the presence of protons. Upon TBA-H_2_PO_4_ addition at the same molar ratio (0.5 mM), a new well-defined peak appears at -0.075V, indicating that the phosphomolybdate complex is formed in the organic solvent. We note that the relatively weak peak intensities are related to the low amount of POM used (1mg) to mimic the same reaction conditions in the PE formulation.

1. **Further characterization of POM@PMO-PE**


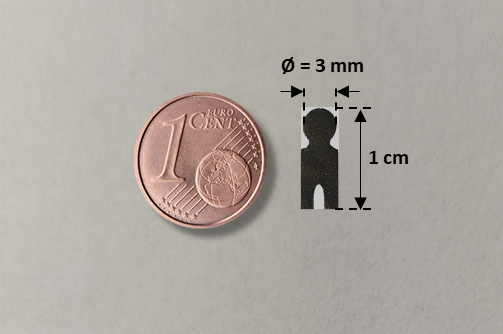


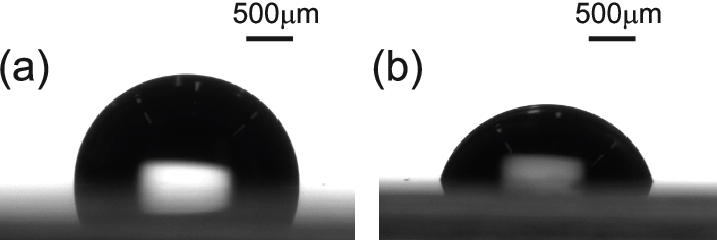
**Figure S6.** Real dimensions of PE electrode

**Figure S7.** Contact angle measured with a Kraüss (Hamburg, Germany) drop shape analyzer (model DSA25) adding water at the electrode surface (25 ul drop). The presence of PMO (b) in Mo-PE (a) modifies the surface wettability.

1. **Phosphate detection using POM@PMO-PE based on PMO-NH_2_**

The response obtained with POM@PMO-PE based on PMO-NH_2_ suggests that the driving force of the interaction between POM and PMO is not only the charge interaction. In fact, even nanoarchitectures functionalized with amino groups would show positive charges in acidic operating conditions. The zeta potential of ca +15 mV suggests a marked hydrophilic character of these nanospheres. We, therefore, argued that the low affinity of these amino-functionalized PMOs for both the organic phase of the electrode (graphite) and of the molybdate derivative may have produced an uneven distribution of the POM@PMO hybrids in the PE formulation, which gives less reliable results in phosphate measurement.

1. **POM@PMO-PE based on 2:1 POM:PMO ratio**

The POM amount has already been optimized to 1 mg in our last work (Figueredo et al., 2021) and selected based on the highest sensitivity of the POM-PE sensor. Keeping the same POM quantity, we investigated the 2:1 mass ratio of POM:PMO, considering that organosilica particles could load more POM in their porous structure. However, a technical limitation related to the bad dispersibility of organosilica nanoparticles in the electrode formulation when exceeded 1 mg. The obtained film was uneven, with a bad distribution of the nanocomposite, which altered the sensors’ batch-to-batch reproducibility.

In addition, the obtained data revealed a decline in the correlation between phosphate concentration and the SWV signal (Figure S8). We then assumed that an increased octamolybdate amount would have the same effect as in the previous POM-PE sensor, i.e., a decrease in the sensor’s sensitivity towards phosphate.

**Figure S8.** SWV response of POM@PMO-PE sensor made with 2:1 of POM:PMO mass ratio showing a broad oxidation peak at -0.03 V and a weak sensitivity to increasing target concentrations in standard solutions.

1. **Step-by-step experimental protocol for real sample analysis**

- Take a volume of 1-2mL of the water to analyze (Ideally, water should be sampled as recommended by EPA-certified method).
- If needed, filtrate the sample using a 0.2 μm syringe filter (cellulose membrane).
- Dilute the filtered sample x2500 times using the acidic electrolyte solution (1M H_2_SO_4_ in 0.1M KCl) to obtain at least 5 mL of diluted sample ready to test.
- Make sure the POM@PMO-PE sensors are pre-activated by incubation in the same electrolyte for one hour before analysis.
- Mount the 3-electrodes electrochemical cell by using the activated PE sensors as the working electrode, an Ag/AgCl electrode as a reference, and a counter electrode of your choice (Carbon, Platinium, etc.)
- Connect the E-cell to any available potentiostat and record the square wave voltammetry using the following parameters: E_Initial_ = -0.5 V , E_Final_ = 0.3 V, Frequency = 25 Hz, E_step_ = 2.5 mV, Amplitude= 0.1 V.
- Analyze the blank electrolyte and then the diluted sample (V= 5 mL).
- Record the maximum peak current value in μA at about -0.06 V to obtain I_0_ for the blank and I for the sample.
- Calculate the relative current variation in μA by subtracting any background noise (I_corrected_ = I - I_0_).
- Extrapolate the obtained value of relative current to the calibration curve and determine the corresponding phosphate concentration in μM. (Ps: The user may need to trace a new calibration curve for more accurate results).
- Multiply the found phosphate concentration by the dilution factor x2500 to obtain the real value in the parent sample before dilution.

**References**

1. Day, V. W.; Fredrich, M. F.; Klemperer, W. G.; Shum, W. Synthesis and Characterization of the Dimolybdate Ion, Mo2O72-. J Am Chem Soc 1977, 99 (18), 6146–6148. https://doi.org/10.1021/JA00460A074.
2. Figueredo F., Girolametti F., Aneggi E., Lekka M., Annibaldi A., Susmel S., Plastic Electrode Decorated with Polyhedral Anion Tetrabutylammonium Octamolybdate [N(C4H9)4]4 Mo8O26 for NM Phosphate Electrochemical Detection. *Analytica Chimica Acta* **2021**, 1161, 338469. doi.org/10.1016/j.aca.2021.338469.
3. F. Albert Cotton; Geoffrey Wilkinson; Carlos A. Murillo; Manfred Bochmann. Advanced Inorganic Chemistry, 6th Edition; 1999.
4. Guan, B.; Cui, Y.; Ren, Z.; Qiao, Z.; Wang, L.; Liu, Y.; Huo, Q. Highly Ordered Periodic Mesoporous Organosilica Nanoparticles with Controllable Pore Structures. Nanoscale 2012, 4 (20), 6588. <https://doi.org/10.1039/c2nr31662e>.
5. M. Fanelli, F. Girolametti, C. Truzzi, S.Illuminati, B. Ajdini, S. Susmel, M. Celussi, J. Šangulin, A. Annibaldi. Impact of Depuration Plants on Nutrient Levels in the North Adriatic Sea. Water 2022, 14, 1930 <https://doi.org/10.3390/w14121930>
6. A. Motealleh, R. De Marco, N.S. Kehr. Stimuli-responsive local drug molecule delivery to adhered cells in a 3D nanocomposite scaffold. J. Mater. Chem. B, 2019, 7, 3716.
7. B. Guan, Y. Cui, Z. Ren, Z. Qiao, L. Wang, Y. Liua and Q. Huo. Highly ordered periodic mesoporous organosilica nanoparticles with controllable pore structures. Nanoscale, 2012, 4, 6588.
8. Nagul, E. A.; McKelvie, I. D.; Worsfold, P.; Kolev, S. D. The Molybdenum Blue Reaction for the Determination of Orthophosphate Revisited: Opening the Black Box. *Analytica Chimica Acta* **2015**, 890, 60–82. [doi.org/10.1016/j.aca.2015.07.030](https://doi.org/10.1016/j.aca.2015.07.030).
9. Cindrić M., Veksli Z., Kamenar B., Polyoxomolybdates and Polyoxomolybdovanadates – from Structure to Functions: Recent Results. *Croatica Chemica Acta* **2009**, 82, 345-362
10. Zhang S., Lu Y., Sun X-W., Li Z., Dang T-Y., Zhang Z., Tiana H-R., Liu S-X., Purely inorganic frameworks based on polyoxometalate clusters with abundant phosphate groups: single-crystal to single-crystal structural transformation and remarkable proton conduction. ***Chem. Commun.****,* **2020**,**56**, 391-394. [doi.org/10.1039/C9CC08696J](https://doi.org/10.1039/C9CC08696J)
11. [Zhai](https://pubmed.ncbi.nlm.nih.gov/?term=Zhai%20L%5BAuthor%5D) L. Li H., Polyoxometalate–Polymer Hybrid Materials as Proton Exchange Membranes for Fuel Cell Applications. [*Molecules*.](https://www.ncbi.nlm.nih.gov/pmc/articles/PMC6803900/) **2019**, 24, 3425. doi: [10.3390/molecules24193425](https://doi.org/10.3390%2Fmolecules24193425)
12. Surface Wetting: Characterization, Contact Angle, and Fundamentals. Kock-Yee Law, Hong Zhao, **2016**, 1^st^ edition, Springer, ISBN: 978-3-319-25214-8
13. M. F. Altahan, E.P. Achterberg, A. G. Ali, M. Abdel-Azzem, NaOH Pretreated Molybdate-Carbon Paste Electrode for the Determination of Phosphate in Seawater by Square Wave Voltammetry with Impedimetric Evaluation, J. Electrochem. Soc., **2021**, 168, 127503, https://doi.org/10.1149/1945-7111/ac3b03
14. M. B. Arvas, O. Gorduk , M. Gencten, Y. Sahin, Preparation of a novel electrochemical sensor for phosphate detection based on a molybdenum blue modified poly(vinyl chloride) coated pencil graphite electrode, **2019**, Anal. Methods, 11, 3874-3881, DOI: 10.1039/C9AY01275C
15. S. Cinti, D.Talarico, G. Palleschi, D. Moscone, F. Arduini, Novel reagentless paper-based screen-printed electrochemical sensor to detect phosphate, **2016**, Anal. Chim. Acta, 919, 78-84, <https://doi.org/10.1016/j.aca.2016.03.011>
16. J. Jońca, M. Comtat, V. Garçon, In Situ Phosphate Monitoring in Seawater: Today and Tomorrow. In: Mukhopadhyay, S., Mason, A. (eds) Smart Sensors for Real-Time Water Quality Monitoring. Smart Sensors, Measurement and Instrumentation, vol 4. Springer, Berlin, Heidelberg. https://doi.org/10.1007/978-3-642-37006-9_2
